# Smurf2 inhibition enhances chemotherapy and radiation sensitivity in non-small-cell lung cancer

**DOI:** 10.1038/s41598-022-14448-8

**Published:** 2022-06-16

**Authors:** Kunal R. Chaudhary, Connor J. Kinslow, Haiying Cheng, Jose M. Silva, Jiyang Yu, Tony. J. Wang, Tom K. Hei, Balazs Halmos, Simon K. Cheng

**Affiliations:** 1 Department of Radiation Oncology, Columbia University College of Physicians and Surgeons, New York, NY USA; 2 Department of Oncology, Albert Einstein College of Medicine of Yeshiva University/Montefiore Medical Center, Bronx, NY USA; 3 Icahn School of Medicine at Mount Sinai, New York, NY USA; 4 Department of Computational Biology, St. Jude Children’s Research Hospital, Memphis, TN USA; 5 Division of Hematology/Oncology, Herbert Irving Comprehensive Cancer Center, New York Presbyterian Hospital–Columbia University Medical Center, New York, NY USA; 6 Department of Radiation Oncology, New York Presbyterian Hospital, Columbia University Medical Center, New York, NY 10032 USA

**Keywords:** Non-small-cell lung cancer

## Abstract

Lung cancer has been the most common cancer worldwide for several decades. The outcomes of patients with locally advanced lung cancer remain dismal, and only a minority of patients survive more than 5 years. However, tumor therapeutic resistance mechanisms are poorly studied. Identification of therapeutic resistance pathways in lung cancer in order to increase the sensitivity of lung tumor cells to therapeutic agents is a crucial but challenging need. To identify novel genes that modulate the response to platinum-based therapy, we performed a genome-wide high-throughput ribonucleic acid interference (RNAi) screen via transfection of human lung cancer (PC9) cells with a viral short hairpin RNA (shRNA) library. We further validated a potential target via 3-(4,5-dimethylthiazol-2-yl)-2,5-diphenyltetrazolium bromide (MTT) and clonogenic survival assays on PC9 and A549 lung tumor cells transfected with small interfering RNAs (siRNAs) to successfully downregulate protein expression and then treated with increasing doses of cisplatin or X-ray radiation. We determined protein expression by immunohistochemistry (IHC) after chemoradiotherapy and analyzed gene expression-based survival outcomes in two cohorts of human non-small-cell lung cancer (NSCLC) patients. The screen identified several targets involved in epithelial-to-mesenchymal transition (EMT), including Smurf1, Smurf2, YAP1, and CEBPZ, and glycolytic pathway proteins, including PFKFB3. Furthermore, we found that the small molecule proteasome inhibitor bortezomib significantly downregulated Smurf2 in lung cancer cells. The addition of bortezomib in combination with cisplatin and radiation therapy in PC9 and A549 cells led to an increase in deoxyribonucleic acid (DNA) double-strand breaks with increased numbers of γ-H2AX-positive cells and upregulation of apoptosis. Finally, we found that Smurf2 protein expression was upregulated in situ after treatment with cisplatin and radiation therapy in a relevant cohort of patients with stage III NSCLC. Additionally, Smurf2 gene expression was the strongest predictor of survival in patients with squamous NSCLC after chemotherapy or chemoradiotherapy. We successfully identified and validated Smurf2 as both a common modulator of resistance and an actionable target in lung cancer. These results suggest the urgent need to investigate clinical Smurf2 inhibition via bortezomib in combination with cisplatin and radiation for patients with locally advanced NSCLC.

## Introduction

Lung cancer is the most common malignancy, and 80–85% of all lung cancers are non-small-cell lung cancer (NSCLC)^1^. Lung cancer is also the leading cause of cancer-related death worldwide, accounting for more than 1.3 million deaths annually^1^. Despite significant advances with the introduction of immunotherapy with checkpoint blockade, platinum-based therapy remains an essential treatment for most patients with advanced lung cancer^2^. Concurrent chemoradiotherapy (chemoRT) can play an important role in the treatment of patients with locally advanced NSCLC. However, despite progress in the past few decades, current therapeutic regimens have only limited efficacy, and patient outcomes remain dismal, with a 5-year overall survival rate of only 20%^1^. Pathways of treatment failure related to tumor resistance to chemotherapy and radiotherapy are poorly studied^3,4^. Epithelial-to-mesenchymal transition (EMT)^5^, apoptosis suppression, elevated and sustained cell proliferation^6^, and upregulation of the deoxyribonucleic acid (DNA) damage response have been identified as some of the major therapeutic resistance mechanisms^7^. The cytotoxic effects of both radiation and platinum chemotherapy are mediated through DNA damage, and alterations that increase the efficiency of DNA repair may participate in a common mechanism of resistance to both therapies.

Ubiquitin modification, which controls a wide range of cellular functions by shuttling proteins into regulatory complexes, orchestrates multiple crosstalk responses to DNA damage^8^. Specific E3 Ubiquitin Protein Ligase 2 (Smurf2) is an E3 ubiquitin ligase that mediates targeted ubiquitin tagging and has pleiotropic effects on the DNA damage response, maintenance of genomic stability, chromatin modifications and cell cycle control^9,10^. Interestingly, Smurf2 has been implicated in seemingly contradictory roles as a pro-oncogenic factor and as a tumor suppressor. Smurf2 has been shown to regulate the TGFβ pathway^9^ and proteins involved in the control of cell cycle progression, including NEDD9-Aurora A^11,12^, RhoA^12^, KLF2 and KLF5^13,14^, supporting its role in tumorigenesis. Moreover, abnormally high expression of Smurf2 has been reported to occur in pancreatic, renal, breast and esophageal squamous cell carcinomas^15,16^. In contrast, global genomic ablation of Smurf2 in mouse studies has been found to lead to a predisposition to a wide spectrum of tumors, suggesting that Smurf2 may also have an opposing role as a novel tumor suppressor^10,17^. Although early loss of Smurf2 in mouse embryonic fibroblasts (MEFs) increases the number of DNA-damaged γ-H2AX–positive cells resulting from etoposide chemotherapy, Smurf2^−/−^ MEFs exhibit less apoptosis and have higher viability than wild-type MEFs, which leads to accumulation of unrepaired DNA damage and genomic instability^17^. In tumors, increased cellular γ-H2AX is typically used as a biomarker for assessment of DNA damage, the chemotherapeutic response and radiation sensitivity. However, the role of Smurf2 in the DNA damage response in tumors and how it interacts with cancer therapies, such as chemotherapy and radiation, are unclear.

To identify therapeutic resistance pathways in NSCLC, we previously performed an unbiased genome-wide high-throughput ribonucleic acid interference (RNAi) screen^18^. Further analysis of the screening results revealed that Smurf2 is a potential novel target that synergizes with cisplatin chemotherapy and radiation to induce cytotoxicity in NSCLC cell lines. Pharmacological inhibition of Smurf2 with bortezomib was associated with increased sensitization to chemoradiation via increased DNA damage and apoptotic cell death. Smurf2 protein expression was increased in human lung tumors in situ after chemoRT, and Smurf2 gene expression was the strongest predictor of survival in a relevant cohort of patients after chemotherapy or chemoRT.

## Materials and methods

### NSCLC cell lines and reagents

The human NSCLC cell lines PC9 and A549 were purchased from the American Type Culture Collection (Manassas, VA). All NSCLC cell lines were maintained in RPMI 1640 medium supplemented with 10% fetal bovine serum (FBS), 100 U/ml penicillin, and 100 μg/ml streptomycin in a humidified incubator at 37 °C in an atmosphere of 5% CO_2_. Bortezomib was purchased from EMD Millipore (Billerica, MA), cisplatin was obtained from Sigma-Aldrich (St. Louis, MO), andsmallinterfering RNAs (siRNAs) and Lipofectamine were purchased from Life Technologies (Rockville, MD).

### Reverse transcription and quantitative reverse transcription-polymerase chain reaction (qRT–PCR)

RNA was extracted using TRIzol reagent (Life Technologies, Rockville, MD) according to the manufacturer’s protocol. Total RNA (2 μg/μl) was reverse-transcribed using a High-Capacity cDNA Reverse Transcription Kit (Applied Biosystems). qRT–PCR was performed in a ViiA™ 7 Real Time PCR system using TaqMan gene expression assays (Life Technologies, Rockville, MD). Glyceraldehyde-3-phosphate dehydrogenase (GAPDH) was used as the reference gene in all qRT–PCR analyses. The normalized transcript level of the target gene (Smurf2) was determined and used to calculate the fold induction in expression compared to the expression in untreated controls.

### Cell culture and transfection conditions

PC9 and A549 cells were cultured in RPMI 1640 medium supplemented with 1% penicillin–streptomycin and 10% FBS. When the cells were 90% confluent, they were transfected with predesigned siRNA oligonucleotides specific for Smurf2 (Life Technologies) using RNAiMAX (Life Technologies, Carlsbad, CA, USA) following the manufacturer’s instructions. Scrambled siRNA (Life Technologies) was used as a nonspecific control oligonucleotide. All siRNAs were transfected at a final concentration of 50 nM for the indicated times. For overexpression experiments, the cDNA expression plasmid pCMV5B-Flag-Smurf2 C716A (Addgene) was transiently transfected into cells using Lipofectamine 2000 transfection reagent according to the manufacturer’s instructions. The empty vector was transfected as a control.

### 3-(4,5-Dimethylthiazol-2-yl)-2,5-diphenyltetrazolium bromide (MTT) cell proliferation/viability assays

PC9 and A549 cells were seeded at a density of 3000 cells per well in triplicate in 96-well plates in 100 µl of culture medium. After 24 h with or without Smurf2 inhibition (either genetically or pharmacologically with bortezomib)^19^, cells were cultured with multiple concentrations of cisplatin (0.5, 1.0, and 1.5 µM) or exposed to radiation (2, 4, and 6 Gy) for 72 h. Cell survival was determined using an MTT assay kit according to the manufacturer’s protocol (Sigma–Aldrich, Schnelldorf, Germany).

### Clonogenic assays

A total of 200 cells were seeded in 6-well plates and allowed to attach overnight in medium supplemented with 10% FBS. The next morning, the medium was replaced, and the cells with or without Smurf2 inhibition (either genetically or pharmacologically with bortezomib)^20^ were treated with multiple concentrations of cisplatin (0.5, 1.0, 1.5, and 2.0 µM) or with different doses of radiation (2, 4, and 6 Gy). The ranges of cisplatin concentrations and radiation doses are similar to clinically relevant concentrations and doses for the treatment of lung cancer^21^. After 72 h, the medium was replaced with medium supplemented only with 10% FBS, and the cells were cultured for another 7–14 days. The colonies were then washed once with PBS and stained with 0.5% crystal violet in 50% methanol and 10% glacial acetic acid for counting. The numbers of surviving colonies (defined as colonies with > 50 cells) was determined and is expressed as the relative plating efficiency compared to that of control cells. The data are presented as the mean ± standard error of the mean (SEM) from three independent experiments.

### Annexin V apoptosis assay

Irradiated and nonirradiated PC9 and A549 cells were cultured with different concentrations of cisplatin and bortezomib for 72 h for cell cycle analysis or 48 h for DNA damage analysis. Apoptotic cell death was assessed by double staining with fluorescein isothiocyanate (FITC)-conjugated Annexin V and propidium iodide (PI; Apoptosis Detection Kit II, BD Pharmingen) as previously described^22^. DNA damage was evaluated by staining cells with an anti-H2AX (pS139) antibody (BD Pharmingen). Fluorescence-activated cell sorting (FACS) data were acquired using a BD FACS Canto II (BD Biosciences, San Jose, CA). The mean fluorescence index was calculated using FlowJo software (TreeStar, Ashland, USA). Gating was implemented based on negative control staining profiles.

### Flow cytometric analyses of the cell cycle and DNA damage

Irradiated and nonirradiated PC9 and A549 cells (5 × 10^4^) were cultured with cisplatin and/or bortezomib or with DMSO for 72 h for cell cycle analysis or 48 h for DNA damage analysis. For cell cycle analysis, after incubation for the abovementioned durations, cells were harvested, washed in ice-cold PBS, collected by centrifugation, and fixed with 70% ethanol. DNA was stained with PBS containing PI (50 µg/ml) and RNase A (1 mg/ml) (Sigma). After incubation for 30 min at 4 °C in the dark, the cell cycle distribution was determined using the BD FACS Canto II. To assess DNA damage, after incubation for the abovementioned durations, cells were harvested and labeled with an anti-H2AX (pS139) antibody (BD Pharmingen) for 30 min at 4 °C and analyzed using the BD FACS Canto II. The mean fluorescence index was calculated using FlowJo software (TreeStar, Ashland, USA).

### Western blot analysis

Proteins were extracted from PC9 and A549 NSCLC cells with RIPA Lysis and Extraction Buffer (Life Technologies). The protein concentrations were determined using a Bradford assay. Thirty micrograms of protein was heated for 5 min at 95 °C and separated on 4–12% NuPAGE Bis-Tris Precast Gels (Life Technology), and the separated proteins were electrophoretically transferred to nitrocellulose membranes (PROTRAN©, Whatman) using a Western blotting system (Bio–Rad). Nonspecific binding was blocked by incubation with 5% nonfat dry milk or 5% bovine serum albumin (BSA) in TBS-T buffer for 1 h at room temperature, and the membranes were incubated with primary antibodies at the dilutions recommended by the manufacturers at 4 °C overnight with gentle agitation. After three washes with TBS-T, the membranes were incubated with a horseradish peroxidase (HRP)-conjugated secondary antibody (in 5% nonfat milk or BSA in TBS-T) for 1 h at room temperature with gentle agitation. The membranes were washed with TBS-T three times for 10 min each, and the protein bands were visualized by incubation with enhanced chemiluminescence (ECL) reagents (GE Healthcare Biosciences) for 5 min followed by exposure of the membranes in an LAS 4000 mini imaging system (Fuji) until specific signals were detectable. GAPDH was used as the loading control. The antibody against Smurf2 was purchased from Abcam. The anti-GAPDH antibody was purchased from Cell Signaling Technology (Boston, MA).

### Immunohistochemistry (IHC)

Human tissue was obtained with ethical approval from Columbia University’s Institutional Review Board (IRB) with an approved human subject protocol. All methods were performed in accordance with relevant guidelines and regulations. Formalin-fixed human lung tumor tissue sections were sliced into serial sections with a thickness of 5 μm and deparaffinized prior to antigen retrieval with sodium citrate buffer (10 mM, pH 6.0). Endogenous peroxidase activity was blocked with Dako peroxidase blocking reagent (Dako Corporation, Carpinteria, CA). An anti-Smurf2 (D5) antibody was used at a dilution of 1:200, and incubation was conducted overnight at room temperature. Then, a DakoCytomation LSAB 2 System HRP (Dako Corporation, Carpinteria, CA) was used by adding Biotinylated Link Universal and streptavidin-HRP followed by 3’-diaminobenzidine (DAB) chromogen (Dak Corporation, Carpinteria, CA), and nuclear counterstaining was performed with hematoxylin. Semiquantitative analysis of Smurf2 immunohistochemical staining was performed according to the staining intensity and tumor cell positivity (0 = no staining, 1 = weak staining, 2 = moderate staining, 3 = strong staining).

### Gene expression and survival analysis

Data from The Cancer Genome Atlas (TCGA) Program^23,24^ were collected from cBioPortal^25,26^. We queried the Lung Adenocarcinoma and Lung Squamous Cell Carcinoma Firehouse Legacy databases for American Joint Committee on Cancer (AJCC) stage III cancers. Demographic and clinical characteristics were collected. Radiotherapy data were collected from the TCGA PanCancer databases^27^. Overall survival and disease-free survival were compared between subgroups with low and high Smurf2 expression.

### Statistical analysis

All values are expressed as the mean ± SD from at least triplicate experiments. A value of *p* < 0.05 was considered statistically significant, and significance (*p < 0.05, **p < 0.001, ***p < 0.0001) was determined by a two-tailed Student’s t test in GraphPad Prism version 5.0 software (GraphPad, La Jolla, CA). Survival analyses were carried out using the IBM SPSS Statistics software package (International Business Machines Corporation, Armonk, New York). The median survival times were determined using the Kaplan–Meier method, and significance was assessed using the log-rank test. Univariable and multivariable analyses of both overall survival and disease-free survival were conducted using the Cox proportional hazards model. For presentation of survival analysis data, 95% confidence intervals (95% CIs) are shown next to the corresponding hazard ratios (HRs). Differences with a two-tailed *p* value < 0.05 were considered statistically significant. Demographical and clinical characteristics that approached significance with *p* = 0.10 as a cutoff were included in multivariable analyses.

### Ethics approval and consent to participate

Human tissue was obtained with ethics approval from Columbia University’s IRB committee with an approved human subjects protocol (AAAI0481). Waiver of patient consent was approved by the IRB.

## Results

### Smurf2 was identified as a potential candidate modulator of cisplatin resistance

**Figure 1:**
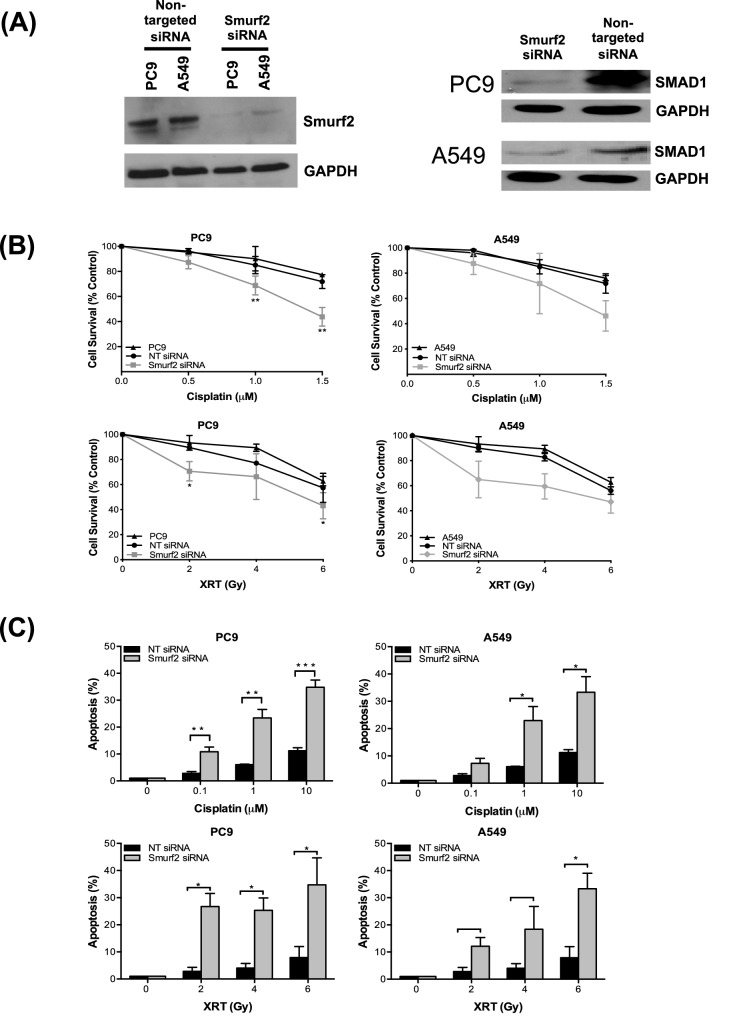
Proliferation and apoptosis analyses of PC9 and A549 NSCLC cells with siRNA-mediated Smurf2 knockdown. Western blot analysis of total Smurf2 and SMAD1 protein levels (**A**) in PC9 and A549 cells after Smurf2 knockdown was confirmed in siRNA-transfected PC9 cells relative to PC9 cells transfected with nontargeting (NT) control siRNA. GAPDH was used as the loading control. Significantly decreased cell survival was observed by MTT assay in Smurf2-knockdown PC9 cells compared to NT siRNA-transfected cells after treatment with increasing concentrations of cisplatin and doses of radiation (RT) (**B**). Quantitative determination of apoptosis in siRNA-mediated Smurf2-knockdown vs. NT siRNA-transfected tumor cells by flow cytometry after treatment with increasing concentrations of cisplatin and doses of radiation (**C**). PC9 and A549 cells were colabeled with FITC-conjugated annexin V (AV) and PI. *p < 0.05; **p < 0.01.

Follow-up analysis of data from our genome-wide high-throughput RNAi screen^18^ indicated that Smurf2 short hairpin RNAs (shRNAs) were depleted 82-fold in the cisplatin screen group compared to the control group, suggesting that the Smurf2 pathway may be a potential novel therapeutic resistance pathway. Smurf2 has been shown to be upregulated in tumors. It promotes metastasis by enhancing cell migration and invasiveness by specifically upregulating the expression of N-cadherin, which is involved in EMT, in a TGF-β/Smad-independent manner^16^. Fukuchi et al. showed that overexpression of Smurf2 correlates with a high invasion depth, lymph node metastasis, a poor survival rate and poor prognosis in patients with esophageal squamous cell carcinoma^15^. To determine the role of Smurf2 in lung cancer cell proliferation and resistance to cisplatin chemotherapy, we first efficiently knocked down Smurf2 protein expression in PC9 and A549 NSCLC cells using three individual siRNAs targeting Smurf2 (Fig. 1A). One potential concern in utilizing siRNAs for phenotypic analysis is that siRNAs may not affect the intended pathway and may have off-target effects. To address this concern, the protein level of SMAD1, a downstream signaling target of Smurf2, was measured. As expected, siRNA targeting Smurf2 greatly reduced the protein level of the Smurf2 downstream target SMAD1, confirming that activation of the Smurf2-SMAD1 pathway was altered (Fig. 1A). After successful establishment of Smurf2 knockdown through siRNA, we evaluated treatment responses in these Smurf2- knockdown PC9 and A549 cells.

### Knocking down Smurf2 sensitized NSCLC PC9 cells to cisplatin and radiation

To determine the synergistic effect of Smurf2 knockdown with cisplatin treatment, an MTT cell survival assay was performed by transfecting 3000 PC9 cells with Smurf2 siRNA in triplicate and then treating the cells with increasing doses of cisplatin (0.5, 1, and 1.5 µM). The survival of Smurf2 siRNA-transfected PC9 and A549 cells treated with cisplatin was significantly reduced compared to that of the control siRNA-transfected cells (Fig. 1B). Moreover, greater apoptosis was detected in Smurf2-knockdown PC9 and A549 cells treated with higher concentrations of cisplatin than in their control counterparts (Fig. 1C), as shown by Annexin V flow cytometric analysis. In addition to evaluating the effect of cisplatin treatment, we further determined the effect of X-ray irradiation (2, 4 and 6 Gy) on Smurf2-knockdown PC9 cells. Consistent with the findings for cisplatin treatment, X-ray irradiation synergized with Smurf2 knockdown, resulting in significantly decreased survival (Fig. 1B) and increased apoptosis (Fig. 1C) in Smurf2 siRNA-transfected cells compared with control siRNA-transfected cells.

### Cotreatment with bortezomib, a pharmacological inhibitor of Smurf2, sensitized NSCLC cells to cisplatin and radiation

**Figure 2:**
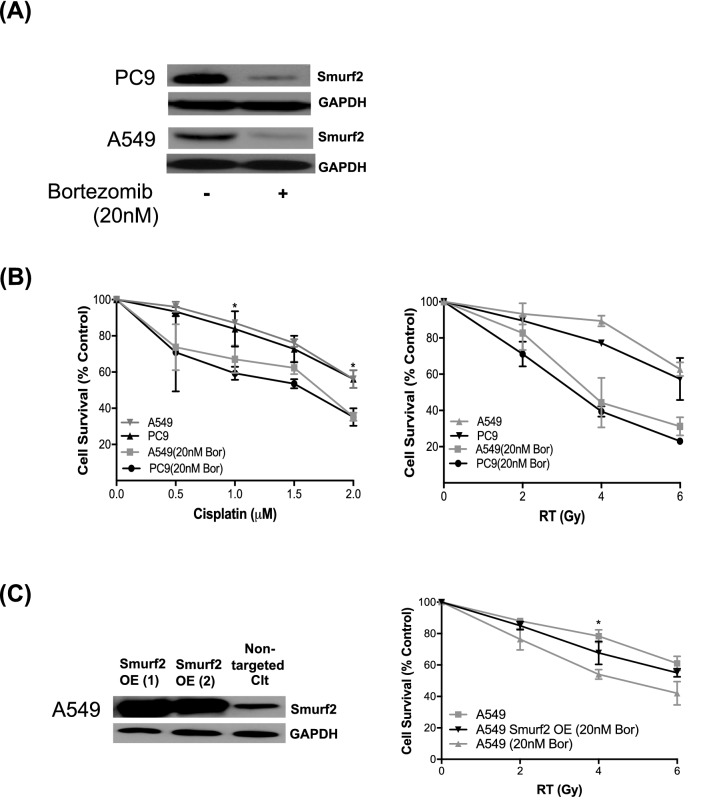
Clonogenic survival analysis and Smurf2 expression in bortezomib-treated PC9 and A549 cells. Western blot analysis showed that 20 nM bortezomib reduced Smurf2 protein expression in PC9 and A549 cells (**A**). Combined treatment with bortezomib (20 nM) plus cisplatin and bortezomib (20 nM) plus radiation in PC9 and A549 cells significantly decreased clonogenic survival compared to that of the corresponding cells treated with cisplatin and radiation alone, respectively (**B**). Overexpression of Smurf2 with two separate targeting plasmid constructs in A549 cells (Smurf2 OE 1, 2) compared to wild-type expression of Smurf2 in control A549 cells (nontargeting control). Overexpression of Smurf2 rescued A549 cells from the toxic effects of radiation and bortezomib treatment, as evaluated by clonogenic assays (**C**).

Bortezomib (also called PS-341 or Velcade; Millennium Pharmaceuticals, Cambridge, MA) is a potent selective reversible small molecule proteasome inhibitor; it is already approved for the treatment of patients with multiple myeloma and is under clinical trials for various malignancies, including NSCLC^28^. Bortezomib sensitizes solid tumors to apoptosis^29^ and is a potent radiosensitizer and modulator of apoptotic sensitivity in preclinical models^30,31^. Compared with radiation alone, pretreatment with bortezomib and subsequent radiation increases the apoptosis and decreases the growth and clonogenic survival of myeloma and colon cancer cells^32,33^. Possible antitumor mechanisms triggered by bortezomib alone or in combination with other agents are differential effects on pro- and antiapoptotic members of the Bcl-1 family, downregulation of XIAP and survivins, upregulation of death receptors (DR4&5), and induction of mitotic catastrophe^34^. However, the molecular mechanisms underlying bortezomib-mediated enhancement of sensitization to chemotherapy and radiation, particularly in NSCLC, remain largely uncharacterized. Bortezomib has been shown to inhibit the proliferation of prostate cancer cells by reducing Smurf2 expression^19^. Considering this finding, we sought to determine whether bortezomib can decrease Smurf2 expression in NSCLC cells and to further examine the effects of pharmacological inhibition of Smurf2 signaling with bortezomib in combination with cisplatin and radiation. Treatment with bortezomib led to reduced protein expression of Smurf2 in PC9 and A549 cells (Fig. 2A). Next, PC9 and A549 cells were treated with bortezomib (20 nM) alone, bortezomib (20 nM) plus cisplatin or bortezomib (20 nM) plus radiation and were analyzed after an incubation period of 72 h. Bortezomib synergized with cisplatin and radiation, and dose-dependent cytotoxicity was observed (Fig. 2B). Given that bortezomib may have pleomorphic effects in addition to Smurf2 downregulation, we sought to determine whether bortezomib synergizes with radiation in a Smurf2-dependent manner. We constitutively overexpressed the Smurf2 protein in A549 cells by transient transfection of plasmids expressing human Smurf2 (Fig. 2C). Smurf2-overexpressing A549 cells were exposed to different doses of radiation in combination with 20 nM bortezomib. Overexpression of Smurf2 in A549 cells attenuated the cytotoxicity observed in cells subjected to combined treatment with radiation and bortezomib compared to that in control A549 cells (Fig. 2C). This finding suggests that bortezomib does indeed synergize with radiation in a Smurf2-dependent manner.

### Combined treatment with bortezomib, cisplatin and radiation enhanced DNA damage and upregulated apoptosis in NSCLC cells

**Figure 3:**
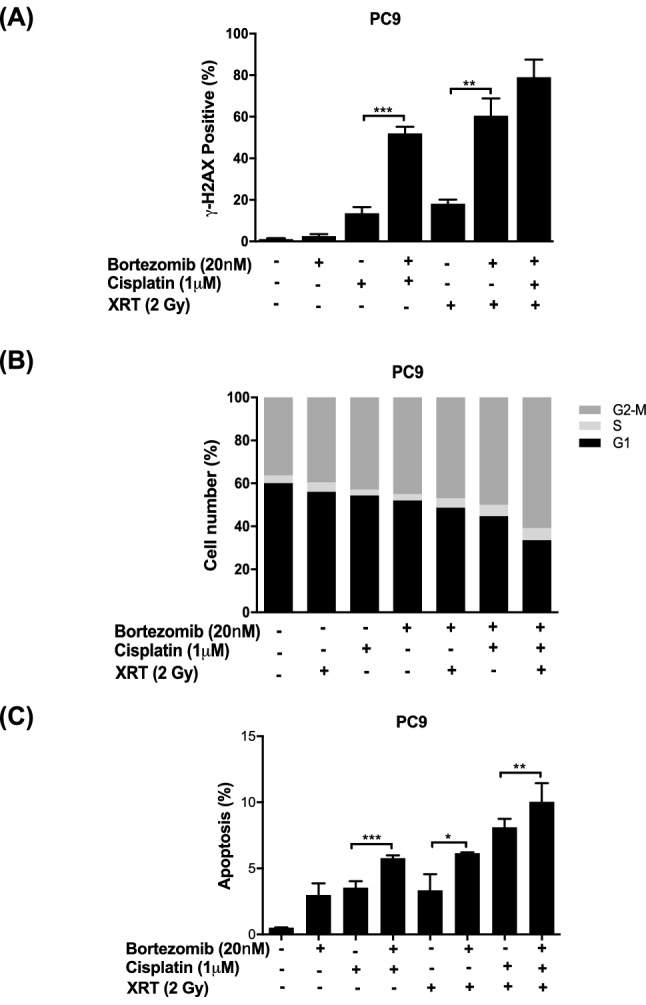
Combined treatment with bortezomib, cisplatin and radiation results in enhanced DNA damage, cell cycle arrest and enhanced apoptosis in NSCLC cells. Smurf2 inhibition with bortezomib significantly enhanced DNA damage, as shown by the significantly increased percentage of γ-H2AX-positive NSCLC cells after treatment with bortezomib plus cisplatin or radiation compared to treatment with bortezomib, cisplatin, or radiation alone (**A**). In PC9 cells, combined treatment with bortezomib plus cisplatin plus radiation induced G2/M-phase arrest (**B**), which was associated with increased apoptosis (**C**).

To gain insights into the mechanisms underlying the synergism of bortezomib, cisplatin and radiation in NSCLC cells, we next investigated the effects of these treatments on apoptosis. Both cisplatin and radiation cause cytotoxic DNA damage, thereby leading to cell death. To determine whether bortezomib acts by enhancing DNA damage, we measured its effects on γ-H2AX, a biomarker for double-strand DNA breaks. Bortezomib alone did not cause DNA damage (Fig. 3A). Interestingly, the numbers of γ-H2AX-positive cells were significantly higher in the groups treated with bortezomib plus cisplatin or bortezomib plus radiation than in the groups treated with either cisplatin or radiation alone in both PC9 cells (Fig. 3A) and A549 cells (data not shown). Furthermore, compared to treatment with cisplatin, radiation or bortezomib alone, combined treatment with bortezomib plus cisplatin, bortezomib plus radiation or bortezomib plus chemoradiation increased G2/M cell cycle arrest in both PC9 cells (Fig. 3B) and A549 cells (data not shown). As shown in Fig. 3C, flow cytometric analysis revealed increased apoptosis induction in cells treated with bortezomib plus cisplatin, bortezomib plus radiation, or bortezomib plus chemoradiation compared with cells treated with cisplatin, radiation or chemoradiation alone. These results suggest that bortezomib can overcome therapeutic resistance and sensitize NSCLC cells to both cisplatin and radiation by enhancing DNA damage and apoptosis via downregulation of Smurf2. Therefore, bortezomib has promising antitumor activity in combination with cisplatin and radiation for lung cancer treatment.

### Immunohistochemical analysis of Smurf2 expression in primary human NSCLC

**Figure 4:**
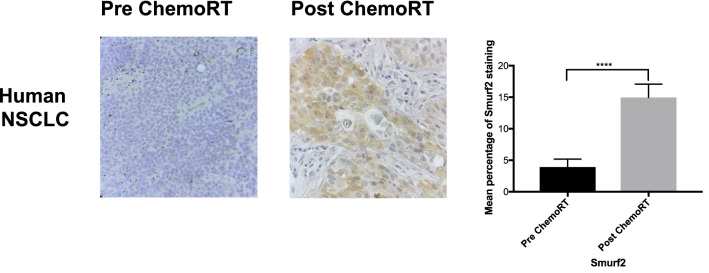
ChemoRT upregulates Smurf2 protein expression in tumor cells in patients with stage III NSCLC. Smurf2 IHC revealed significantly higher Smurf2 protein expression in the post-chemoRT-patient lung tumor samples (n = 8) than in the pre-chemoRT tumor samples (n = 7). Representative examples are shown.

Tolerance to anticancer therapies can be acquired by alterations in the expression of Resistance pathway components. To determine whether Smurf2 protein expression is upregulated in human lung tumors treated with chemoRT, we analyzed tumor samples from patients with stage III NSCLC before any treatment (pre-chemoRT group, *n* = 7) and after cisplatin chemotherapy and lung radiation followed by surgery (post-chemoRT group, n = 8). Significantly enhanced upregulation of Smurf2 protein expression was found in post-chemoRT lung tumor samples (Fig. 4) compared to pre-chemoRT tumor samples. To better quantify the expression difference, five randomly chosen microscopic fields per section were imaged, and Smurf2 expression was measured quantitatively as the mean stained area. The immunostaining scores revealed a statistically significant difference between the pre-chemoRT and post-chemoRT primary lung tumors.

### Correlation between Smurf2 mRNA expression and survival in NSCLC

**Figure 5:**
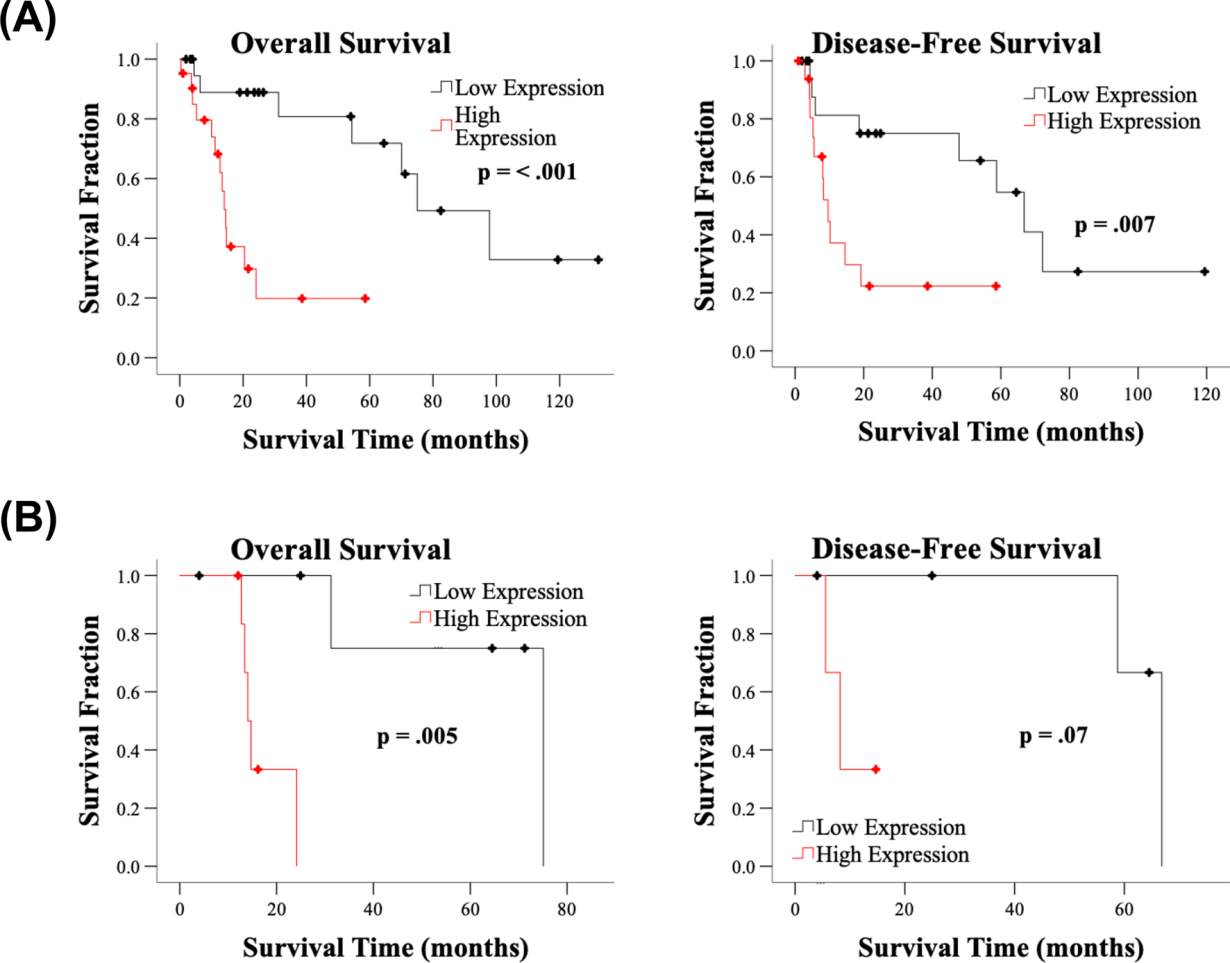
Smurf2 gene expression predicts tumor progression and mortality in patients with stage III squamous NSCLC. Overall survival and disease-free survival were significantly reduced in the high-Smurf2 expression groups of patients with stage III disease treated with chemotherapy (n = 85) (**A**) or treated with combined radiotherapy and chemotherapy (n = 25) (**B**). The black and red lines indicate the 1st and 4th quartiles of expression, respectively. See Table 1 for the associated HRs and 95% CIs.

To determine whether increased Smurf2 expression predicts tumor progression and poorer survival in patients who receive chemoRT, we selected a cohort of patients with stage III NSCLC, for whom platinum-based chemotherapy or chemoRT is the standard of care, from the TCGA database^35^. We also collected demographic and clinical characteristics, including age, sex, race, AJCC tumor stage, AJCC nodal stage, surgical margin, adjuvant targeted therapy status, and radiotherapy status, to assess potentially confounding effects on survival. Although Smurf2 mRNA expression was not prognostic for patients with adenocarcinoma (data not shown), increased Smurf2 mRNA expression was associated with poorer overall survival (median survival time 14 vs. 75 months for the 4th vs. the 1st quartile, *p* = 0.0005) and disease-free survival (10 vs. 67 months, *p* = 0.007) in patients with squamous cell carcinoma treated with chemotherapy (Fig. 5A). Similar robust effects were seen in the subset of patients who received radiotherapy in addition to chemotherapy (Fig. 5B). Interestingly, Smurf2 expression was the only variable significantly associated with overall survival (HR 5.20 for the 4th vs. the 1st quartile, *p* = 0.001) or disease-free survival (HR 3.77, *p* = 0.008; Table 1) in the cohort. The effects of race, tumor stage, and surgical margin on disease-free survival approached but did not reach statistical significance; these variables were included in the multivariable analysis. In multivariable analysis, Smurf2 expression was the only variable independently associated with significantly decreased disease-free survival (HR 3.77 for the 4th vs. the 1st quartile, *p* = 0.02; Table 2).

**Table 1:** Overall and disease-free survival in patients with stage III squamous NSCLC.

Variable	Reference	Overall survival				Disease-free survival			
		*P* value	HR	95% CI		P Value	HR	95% CI	
				Lower	Upper			Lower	Upper
Age		0.40	1.02	0.98	1.06	0.77	0.99	0.95	1.04
Sex (Female)	Male	0.24	0.65	0.32	1.33	0.32	0.66	0.29	1.51
Race	White	0.41				0.07			
Asian		0.29	2.99	0.39	22.71	0.28	3.11	0.40	23.91
Black		0.58	1.36	0.47	3.94	0.22	1.88	0.69	5.11
NA		0.30	0.69	0.34	1.395	0.06	0.36	0.12	1.04
T stage	T1-T2	0.61				0.23			
T3		0.51	1.26	0.63	2.51	0.09	2.02	0.90	4.54
T4		0.33	1.46	0.68	3.14	0.26	1.79	0.66	4.87
N stage	N0	0.47				0.46			
N1		0.25	1.74	0.68	4.42	0.48	1.44	0.53	3.95
N2		0.76	1.16	0.46	2.95	0.64	0.78	0.28	2.22
N3		0.34	2.20	0.43	11.28	0.98	0.00	0.00	
Surgical margin	R0	0.44				0.23			
R1-R2		0.22	2.15	0.64	7.18	0.89	0.87	0.12	6.54
RX		0.90	0.95	0.42	2.14	0.09	1.98	0.90	4.36
Adjuvant targeted therapy	None	0.28				0.25			
Yes		0.18	0.45	0.14	1.44	0.19	0.51	0.18	1.40
NA		0.90	1.05	0.51	2.15	0.12	0.54	0.25	1.18
Radiation therapy	T1-T2	0.40				0.63			
Yes		0.97	1.01	0.51	2.01	0.77	1.11	0.55	2.26
NA		0.20	1.71	0.76	3.85	0.39	0.41	0.05	3.10
Smurf2 expression	Quartile 1	**0.01**				**0.05**			
Quartile 2		**0.01**	3.47	1.28	9.38	0.35	1.69	0.56	5.08
Quartile 3		0.07	2.37	0.93	6.01	0.47	1.47	0.52	4.10
Quartile 4		**0.001**	5.20	1.93	13.96	**0.008**	3.77	1.41	10.07

R0, R1, R2, and RX surgical margins: no microscopic residual tumor, microscopic residual tumor, macroscopic residual tumor, and unknown residual tumor status, respectively. HR: hazard ratio. CI: confidence interval. NA: not applicable.

Bold text indicates statistical significance.

**Table 2:** Multivariable analysis of disease-free survival in patients with stage III squamous NSCLC.

Variable	Reference	*P* value	HR	95% CI	
				Lower	Upper
Smurf2 expression	Quartile 1	0.16			
Quartile 2		0.16	2.54	0.70	9.29
Quartile 3		0.23	2.08	0.63	6.87
Quartile 4		0.02	3.77	1.21	11.80

Race, T stage, and surgical margin were included in the multivariable analysis.

## Discussion

In this study, we found that Smurf2 participates in a common mechanism for tumor resistance to cisplatin and radiation. We evaluated the effects of Smurf2 knockdown by both genetic ablation (siRNA) and pharmacological inhibition (using the small molecule inhibitor bortezomib, which we also showed downregulates Smurf2) combined with cisplatin chemotherapy and irradiation in two different NSCLC cell lines, PC9 and A549, and observed that Smurf2 knockdown led to significantly reduced cell survival. In addition, bortezomib enhanced the cytotoxicity of cisplatin and radiation by increasing DNA damage in lung tumor cells.

Interestingly, loss of Smurf2 function may have differential effects in nontumor vs. tumor cells. Compared with wild-type MEFs, Smurf2^−/−^ MEFs treated with etoposide chemotherapy exhibit increased DNA damage but decreased apoptosis and increased viability, resulting in increased genomic instability^17^. In contrast to the observations in MEFs, we observed that loss of Smurf2 function enhanced apoptosis and increased cytotoxicity in lung tumor cells (Fig. 1B–C). We speculate that tumor cells have more genetic alterations in DNA repair and proliferation pathways than MEFs, thus enabling more efficient induction of the apoptotic pathway. Indeed, Smurf2 has been suggested to promote cell proliferation by negatively regulating p53 stability and activity by interacting with MDM2 and thereby inhibiting apoptosis in breast tumor cells^36^. We found that in PC9 lung tumor cells, bortezomib synergized with chemoradiation to upregulate the proapoptotic DNA repair molecule C-PARP (data not shown) and cause cell cycle dysregulation or cell cycle arrest at the G2/M phase. This finding further suggests that Smurf2 inhibition may result in a differential cytotoxic response in normal cells vs. tumor cells.

Smurf2 encodes an E3 ubiquitin protein ligase that was originally identified as a mediator of degradation of Smads in the TGF-β/BMP pathway^11,37^. However, it has been increasingly realized that Smurf2 modulates pleomorphic pathways in different cancers. Upregulation of Smurf2 has been shown to be associated with poor prognosis and to promote tumor development in esophageal cancers^15^ and prostate cancers^19^. Elevated levels of Smurf2 expression have also been detected in other cancers, such as pancreatic, renal, and breast cancers^15,16^. Similarly, we found that Smurf2 expression was upregulated after chemoRT in lung tumors and that Smurf2 expression predicted disease progression and mortality after chemoRT. These findings suggest that Smurf2 may be regulated as a resistance mechanism. Indeed, a recent study implicated Smurf2 overexpression as a mechanism of resistance to mitogen-activated protein kinase kinase (MEK) inhibitors in melanoma and found that Smurf2 depletion sensitized melanoma cells to the cytotoxic effects of selumetinib^38^. Combined with our results indicating that Smurf2 inhibition can also sensitize cells to the cytotoxic effects of cisplatin-based chemotherapy and radiation, these previous results indicate that Smurf2 may act as a common modulator of resistance to diverse cytotoxic cancer therapies.

Given bortezomib’s sensitizing ability in various other cancers, several phase I and II clinical trials have been carried out to evaluate bortezomib in combination with standard-of-care platinum-based chemotherapy plus radiation in patients with locally advanced lung cancer^39,40^. In a small phase I trial of 12 patients evaluating the addition of bortezomib to platinum-doublet chemotherapy and concurrent radiotherapy as induction therapy followed by surgical resection in patients with stage III NSCLC, a remarkably high percentage (58%) of patients achieved either a complete pathologic response or greater than 99% tumor necrosis^39^. A phase I/II trial of concurrent bortezomib in combination with paclitaxel, carboplatin, and radiation therapy for stage III NSCLC was recently published; this trial showed a promising 12-month survival rate of 73% and a median overall survival time of 25 months, both of which are higher than the historical control values^40^. This result suggests significant synergy of therapeutic responses with this treatment strategy. Our results suggest that Smurf2 is a potential novel target and indicate a new mechanism of bortezomib-mediated sensitization to cisplatin and radiation therapy via Smurf2 inhibition in lung cancer.

## Conclusions

In summary, our study is the first to indicate the potential role of Smurf2 as a common modulator of resistance and to identify Smurf2 as a potential novel actionable target that can improve responses to both cisplatin and radiation therapy in lung cancer. More effective treatment is needed for this devastating disease, and combination therapies that also target resistance mechanisms seem promising. These encouraging preclinical results give further urgency to the investigation of clinical Smurf2 inhibition via bortezomib in combination with cisplatin and radiation therapy for patients with locally advanced NSCLC.

## Data Availability

The datasets used and/or analyzed during the current study are available from the corresponding author on reasonable request.
